# Response of C57Bl/6 mice to a carbohydrate-free diet

**DOI:** 10.1186/1753-6561-6-S3-P71

**Published:** 2012-06-27

**Authors:** Saihan Borghjid, Richard David Feinman

**Affiliations:** 1 Department of Biology, Molloy College, Rockville Centre, NY 11571, USA; 2 Department of Cell Biology, SUNY Downstate Medical Center, Brooklyn, NY 11203, USA

## 

High fat feeding in rodents generally leads to obesity and insulin resistance whereas in humans this is only seen if dietary carbohydrate is also high, the result of the anabolic effect of poor regulation of glucose and insulin. A previous study of C57Bl/6 mice (Kennedy AR, *et al*: *Am J Physiol Endocrinol Metab* (2007) **262** E1724-1739) appeared to show the kind of beneficial effects of calorie restriction that is seen in humans but that diet was unusually low in protein (5%). In the current study, we tested a zero-carbohydrate diet that had a higher protein content (20%). Mice on the zero-carbohydrate diet, despite similar caloric intake, consistently gained more weight than animals consuming standard chow, attaining a dramatic difference by week 16 (46.1 ± 1.38 g *vs.* 30.4 ± 1.00 g for the chow group). Consistent with the obese phenotype, experimental mice had fatty livers and hearts as well as large fat deposits in the abdomino-pelvic cavity, and showed impaired glucose clearance after intraperitoneal injection. In sum, the response of mice was opposite to that in humans where low carbohydrate diets cause greater weight loss than isocaloric controls. The results suggest that rodent models of obesity may be most valuable in the understanding of how metabolic mechanisms work in ways opposite to their effect in humans.

